# Effects and molecular mechanism of chitosan-coated levodopa nanoliposomes on behavior of dyskinesia rats

**DOI:** 10.1186/s40659-016-0093-4

**Published:** 2016-07-04

**Authors:** Xuebing Cao, Dongzhi Hou, Lei Wang, Sai Li, Shengang Sun, Qineng Ping, Yan Xu

**Affiliations:** Department of Neurology, Union Hospital, Tongji Medical College, Huazhong University of Science and Technology, 1277 Jiefang Avenue, Wuhan, 430022 People’s Republic of China; College of pharmacy, Guangdong Pharmaceutical University, Guangzhou, 510006 People’s Republic of China; Department of Neurology, Weifang People’s Hospital, Weifang, 261000 People’s Republic of China; College of Pharmacy, China Pharmaceutical University, Nanjing, 210009 People’s Republic of China

**Keywords:** Dyskinesia, Levodopa liposomes, ERK1/2, DARPP-32, FosB/ΔFosB

## Abstract

**Background:**

Chitosan, the N-deacetylated derivative of chitin, is a cationic polyelectrolyte due to the presence of amino groups, one of the few occurring in nature. The use of chitosan in protein and drug delivery systems is being actively researched and reported in the literature.

**Results:**

In this study, we used chitosan-coated levodopa liposomes to investigate the behavioral character and the expression of phosphorylated extracellular signal-regulated kinase (ERK1/2), dopamine- and cAMP-regulated phosphoprotein of 32 kDa (DARPP-32) and FosB/ΔFosB in striatum of rat model of levodopa-induced dyskinesia (LID). We found that scores of abnormal involuntary movement (AIM) decreased significantly in liposome group (*P* < 0.05), compared with levodopa group. Levels of phospho-ERK1/2, phospho-Thr34 DARPP-32 and FosB/ΔFosB in striatum decreased significantly in liposome group lesion side compared with levodopa group (*P* < 0.05). However, both of two groups above have significantly differences compared with the control group (*P* < 0.05).

**Conclusion:**

Chitosan-coated levodopa liposomes may be useful in reducing dyskinesias inducing for Parkinson disease. The mechanism might be involved the pathway of signaling molecular phospho-ERK1/2, phospho-Thr34 DARPP-32 and ΔFosB in striatum.

## Background

Levodopa-induced dyskinesia (LID) is difficult to treat, negatively affects quality of life, and increases the treatment costs of Parkinson’s disease (PD) patients [1]. Although levodopa currently represents the most effective treatment for PD patients [2], ameliorating cardinal signs such as bradykinesia, akinesia and rigidity [3], these benefits are in some measure overshadowed by the emergence of LID [4]. In the early stages, PD patients usually experience an acceptable quality of life that is impaired in the advanced stages by the emergence of these involuntary movements that frequently occur at the peak of the levodopa effect [5, 6]. In other words, when the patients experience motor complications such as a shortening motor response and the development of dyskinesia, the delivery of levodopa without inducing dyskinesia becomes increasingly difficult [7, 8].

Extracellular signal-regulated kinases 1 and 2 (ERK1/2) are critical mediators of activity-dependent plasticity as demonstrated by their role in long-term potentiation [8], classical conditioning [9], and memory formation [10]. Phosphorylation of ERK1/2 reflects a balance between the activities of upstream kinases and inactivating phosphatases [11]. Previous studies reported that selective agonists for D2-type DA receptors enhanced ERK1/2 phosphorylation in the DA-denervated striatum [12, 13]. l-DOPA produces pronounced activation of ERK1/2 signaling in the dopamine-denervated striatum through a D1-receptor-dependent mechanism, which was associated with the development of dyskinesia [14]. Phosphorylated ERK1/2 was localized to both dynorphinergic and enkephalinergic striatal neurons, suggesting a general role of ERK1/2 as a plasticity molecule during l-DOPA treatment [14, 15]. In addition, the dopamine- and cAMP-regulated phosphoprotein of 32 kDa (DARPP-32) is abundantly expressed in the medium spiny neurons of the striatum. Phosphorylation catalyzed by cAMP-dependent protein kinase (PKA) converts DARPP-32 into an inhibitor of protein phosphatase-1 [16]. Changes in the state of phosphorylation of DARPP-32 reinforce the behavioral effects produced by stimulation or inhibition of the cAMP pathway [16].

Chitosan, the N-deacetylated derivative of chitin, is a cationic polyelectrolyte due to the presence of amino groups, one of the few occurring in nature. This gives chitosan singular chemical and biological characteristics, such as: biocompatibility [17], antibacterial properties, heavy metal ion chelation ability, gel-forming properties and hydrophilicity [17]. Due to its chemical configuration and to features like abundance, low toxicity, hydrophobicity, biodegradability, biocompatibility and antimicrobial activity, chitosan is employed for the preparation of films, gels, microspheres and microcapsules. It has been used in various areas such as biotechnology, cosmetics, food and pharmaceuticals, as a way to release active compounds, among others [18, 19]. The use of chitosan in protein and drug delivery systems is being actively researched and reported in the literature [20].

In this study, we investigated whether the development of LID might be associated with a critical impairment of the expression of p-ERK1/2, p-Thr34 DARPP-32 and FosB/ΔFosB. To examine our hypothesis, 6-OHDA lesioned rat models of PD were set up and administrated separately with chitosan-coated levodopa liposomes/benserazide and levodopa/benserazide.

## Results

### Behavioral characteristics

**Fig. 1:**
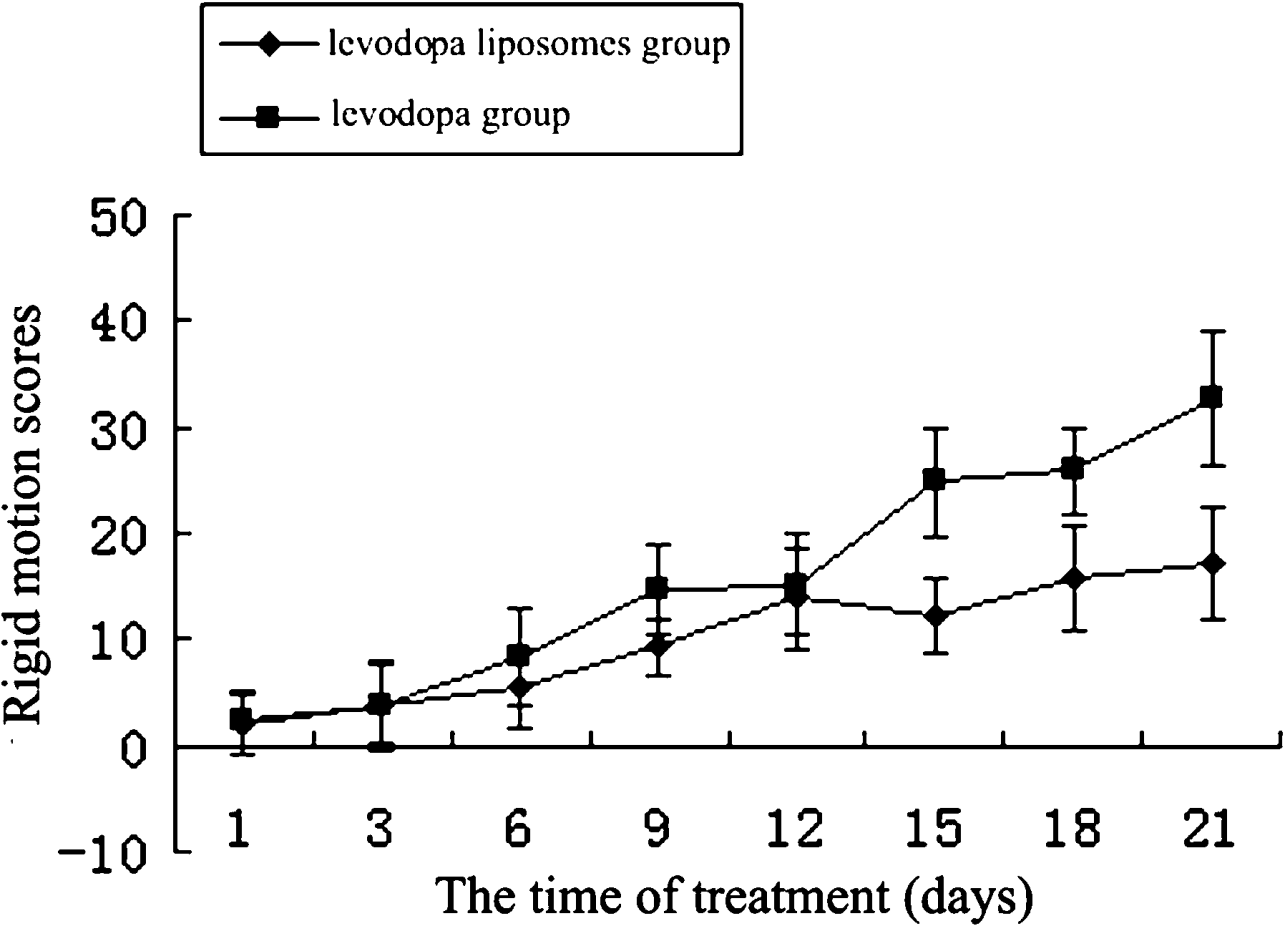
The tendency of rigid motion scores of levodopa and levodopa liposomes groups

**Fig. 2:**
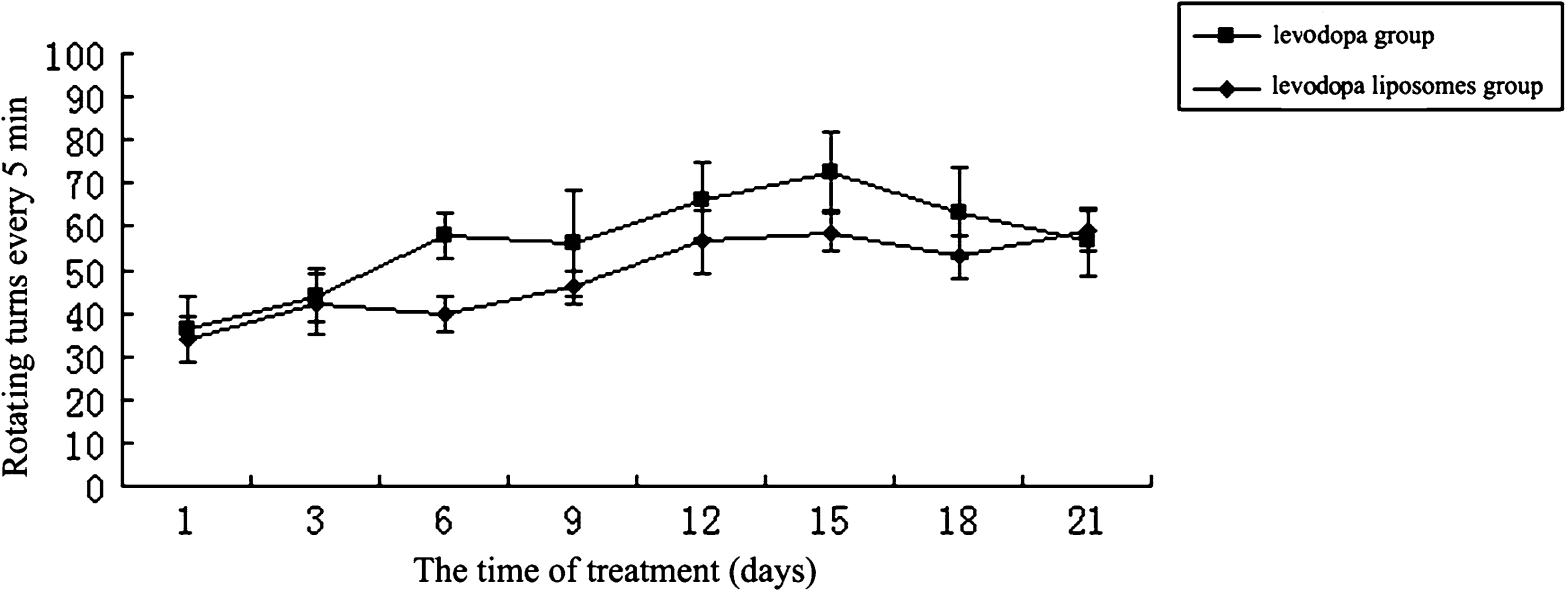
The tendency of rotating turns every 5 min of levodopa and levodopa liposomes groups

There were 18 of total 20 rats and 15 of total 20 rats produced abnormal involuntary movements (AIMs) in 6-OHDA-lesioned after levodopa and levodopa liposomes group, respectively, whereas no AIMs were observed on the saline control group and sham-operated rats. As expected, sham-operated rats did not show AIMs at any time point (data not shown). Most of the levodopa group were shown in severe axial and fore limb AIMs, which scored more than three. However, most of levodopa liposomes group were shown in slight orofacial AIMs, which scored less than two. On the whole, the appearing time of AIMs of levodopa group was earlier than the levodopa liposomes group. The results of LTD rat’s rigid motion scores and rotating turns every 5 min were shown in Figs. 1 and 2, respectively.

### Chronic levodopa induced FosB/ΔFosB expression in damaged lateral striatum

**Fig. 3:**
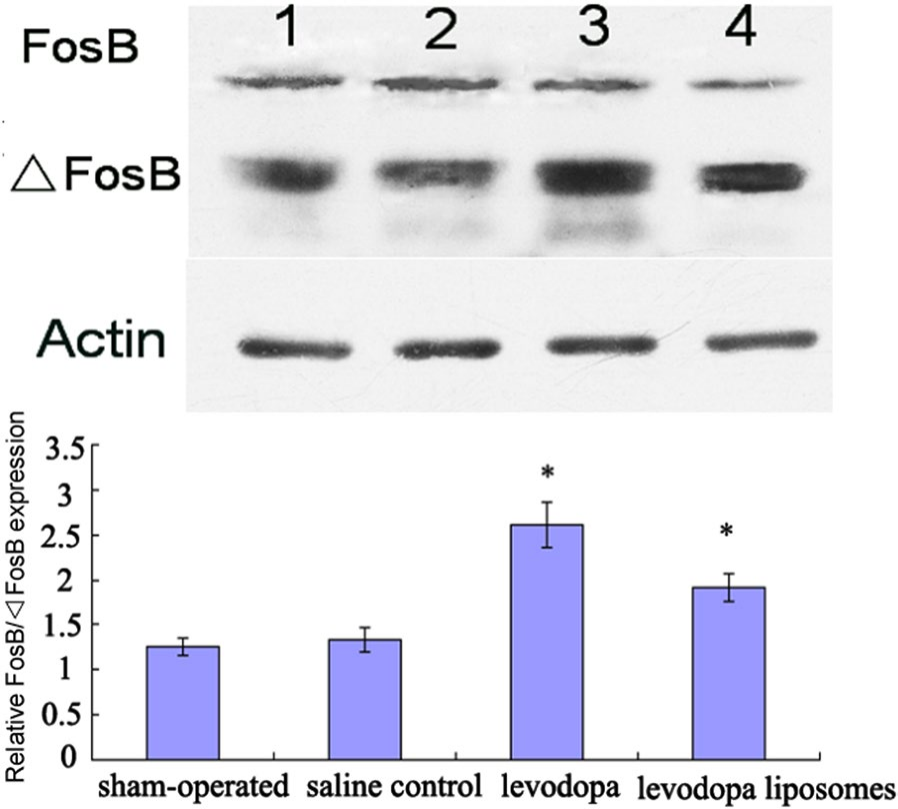
The western blotting results of FosB/ΔFosB expression in the four groups (*1* = sham-operated, *2* = saline control, *3* = levodopa, *4* = levodopa liposomes). *P < 0.05

**Fig. 4:**
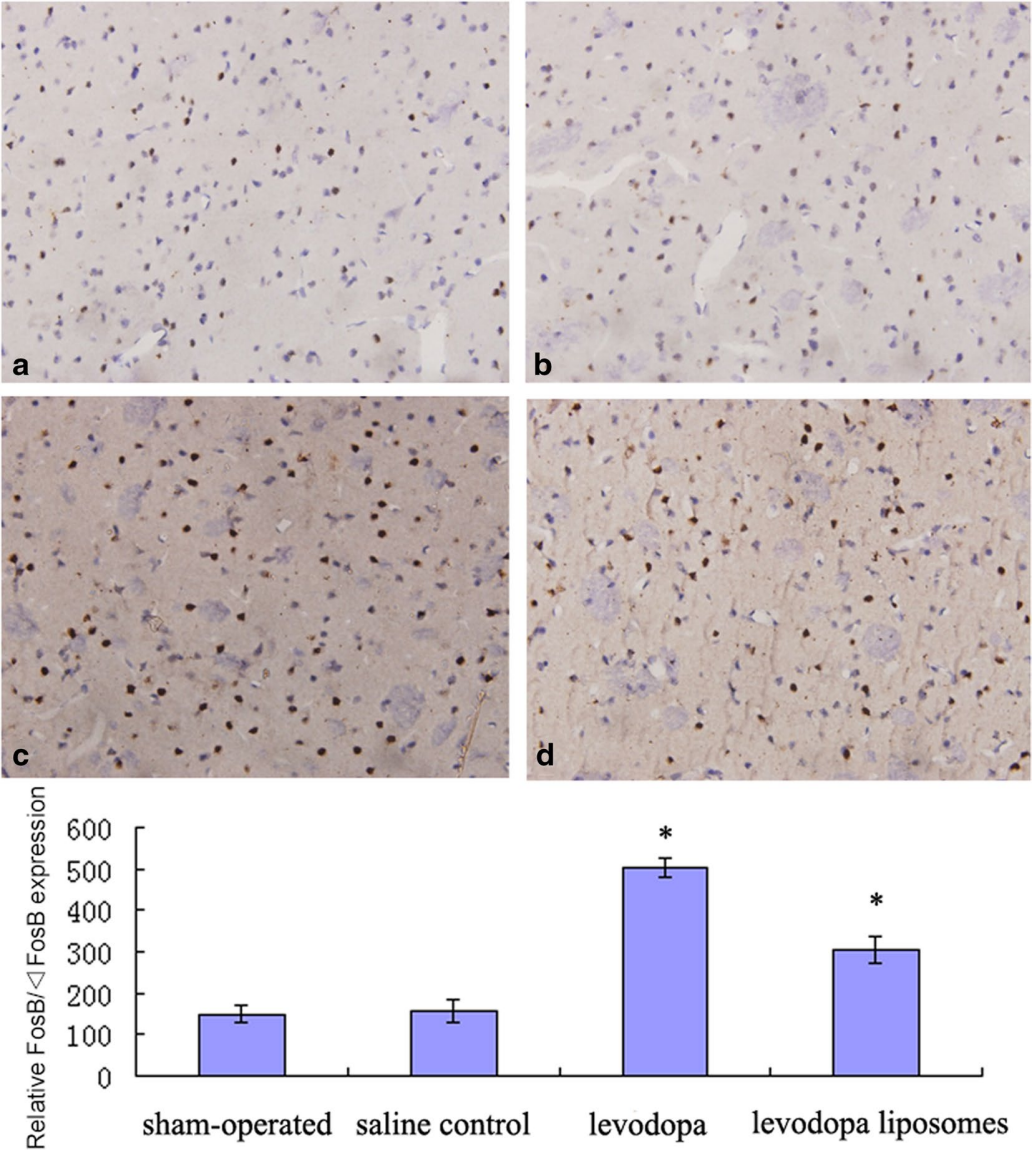
The immunohistochemistry results of FosB/ΔFosB expression in the four groups (**a** = sham-operated, **b** = saline control, **c** = levodopa, **d** = levodopa liposomes). Magnification: ×200 *P < 0.05

Levels of FosB/ΔFosB as quantified by western blotting revealed that chronic levodopa (levodopa group and levodopa liposomes group) had significantly induced FosB/ΔFosB expression (Fig. 3) in 6-OHDA-lesioned rats. Fluorescence in situ hybridization/immunohistochemistry showed that the increasing FosB/ΔFosB positive cells mostly expressed outside of the striatum (Fig. 4).

### Chronic levodopa induced p-ERK1/2 expression in damaged lateral striatum

**Fig. 5:**
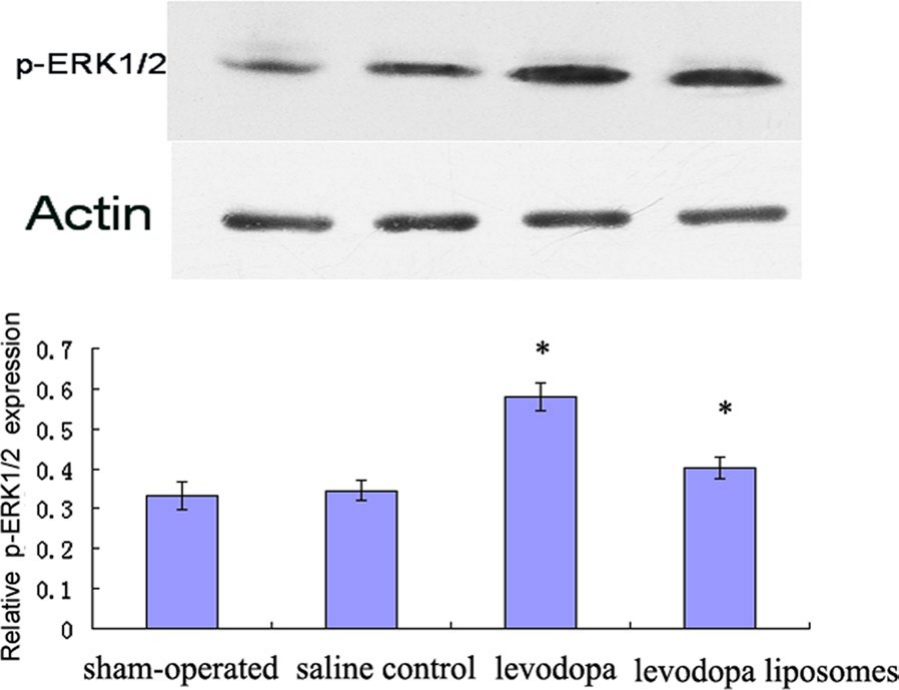
The western blotting results of p-ERK1/2 expression in the four groups (from *left* to *right*): sham-operated, saline control,  levodopa, and levodopa liposomes. *P < 0.05

**Fig. 6:**
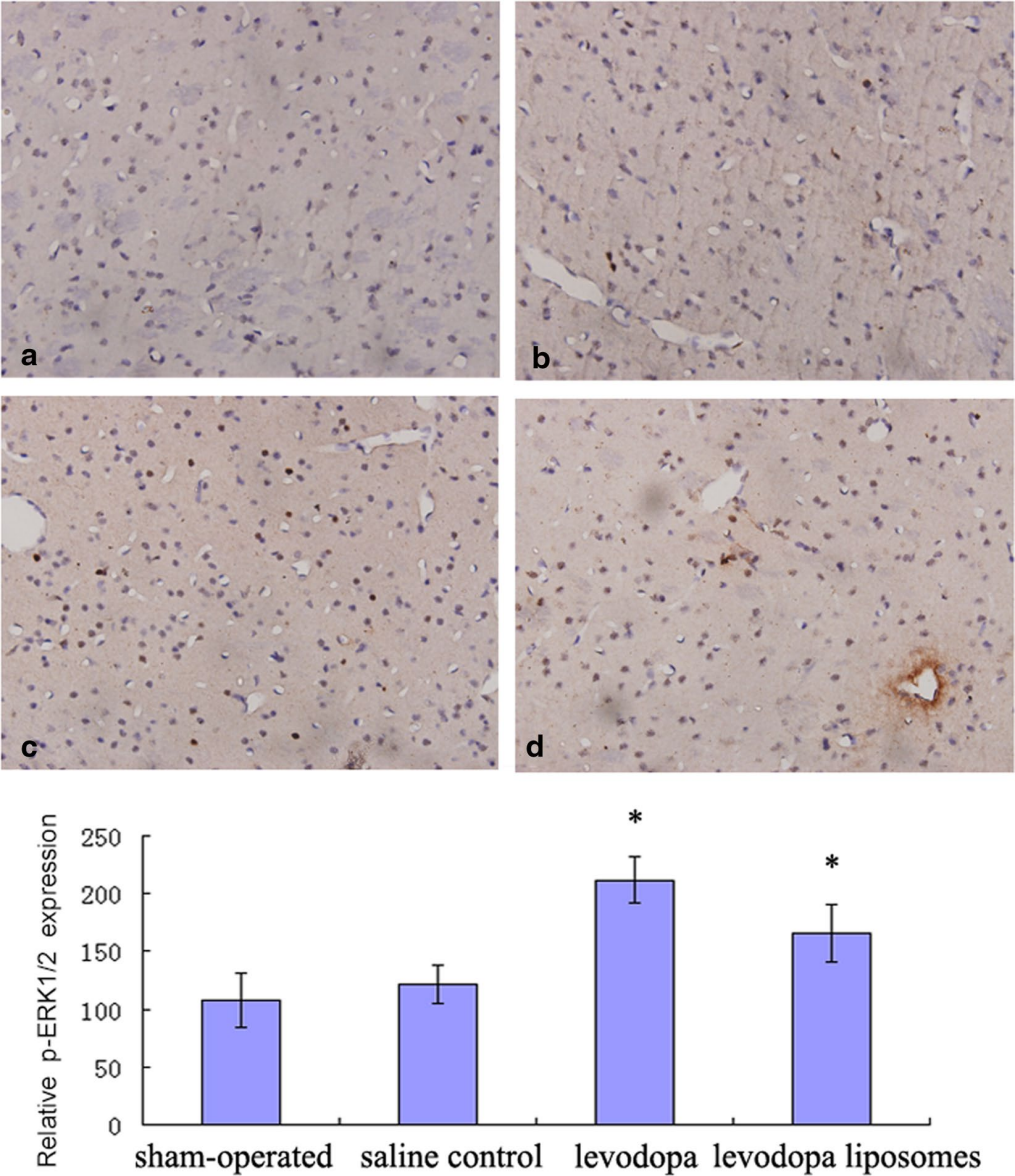
The immunohistochemistry results of p-ERK1/2 expression in the four groups (**a** = sham-operated, **b** = saline control, **c** = levodopa, **d** = levodopa liposomes). Magnification: ×200 *P < 0.05

Levels of ERK1/2 and p-ERK1/2 as quantified by western blotting revealed that chronic levodopa (levodopa group and levodopa liposomes group) had significantly induced p-ERK1/2 expression (Fig. 5) but had no effect on ERK1/2 expression in 6-OHDA-lesioned rats (data not shown). Fluorescence in situ hybridization/immunohistochemistry showed that the increasing p-ERK1/2 positive cells had no obvious differences location distribution (Fig. 6).

### Chronic levodopa induced phospho-Thr34 DARPP-32 expression in damaged lateral striatum

**Fig. 7:**
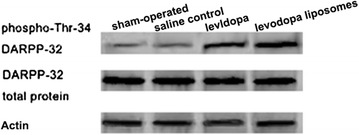
The western blot results of DARPP-32 and phospho-Thr34 DARPP-32 expression in the four groups (sham-operated, saline control, levodopa, levodopa liposomes)

Levels of DARPP-32 and phospho-Thr34 DARPP-32 as quantified by western blotting revealed that chronic levodopa (levodopa group and levodopa liposomes group) had significantly induced phospho-Thr34 DARPP-32 expression but had no effect on DARPP-32 expression in 6-OHDA-lesioned rats (Fig. 7). Western blot assay showed that the increasing phospho-Thr34 DARPP-32 positive cells had no obvious differences location.

## Discussion

LID can be modeled in rats with 6-OHDA unilateral nigro-striatal lesions via chronic administration of low doses of levodopa. In these rats, chronic levodopa induces increasingly severe axial, limb, and orofacial AIMs, which have been extensively characterized by different research groups and validated pharmacologically [21–23]. In present study, we used a unilateral 6-OHDA-lesion PD rats model, which is one of the most frequently used for PD animal model. 6-OHDA must be directional injection because of hardly through the blood brain barrier, which was proofed as the most radical way of damaging nigra striatum pathways [24].

In present study, after chronic levodopa treatment, most animals appeared AIMs and had significantly influence on axial, limb, and orofacial motor function induced by 6-OHDA. There was an obvious individual differences of AIMs symptom which was getting heavier and heavier with the extension of levodopa treatment. This results were the same as the report of [25]. Clinical study shown that about 30–80 % of PD patients could appear LID after chronic levodopa treatment. And with the increase of diseases and the extension of treatment time, the degree of movement disorder and the frequency will increase [26]. In this study, the variation tendency of rotating behavior, rigid motion, and the total AIM score were discrepancy, which was the most significantly in the levodopa group.

Seminal studies evaluating metabolic changes in the basal ganglia have suggested that hyperactivity of the direct pathway sustains dyskinesia [27]. Marked abnormalities in neuronal activity and long-lasting molecular mechanisms prime and/or sustain LID [28]. Among them, striatal FosB/ΔFosB accumulates in PD patients [29] and correlates with LID severity both in rat and monkey models of PD [30]. Molecular interference studies further highlighted a causal link between ΔFosB and LID apparition or expression [31]. In PD patients, the presence of LID is inevitably associated with a decreased duration and/or magnitude of the therapeutic benefit of l-DOPA [32]. In previous study, the selective silencing of FosB/ΔFosB-expressing neurons induced a reduction of LID together with an increased rotational behavior in rats and with an increase in good on time period without changes in disability scores in primates [33]. In present study, the FosB/ΔFosB-expressing of levodopa group and levodopa liposomes group were significantly higher than the saline control group and the sham-operated group. However, the FosB/ΔFosB-expressing of levodopa liposomes group was significantly lower than the levodopa group, which suggested that application of chitosan-coated levodopa nanoliposomes may reduces the different movement disorder in PD treatment, compared to ordinary levodopa tablets.

In chronically l-DOPA treated rats; the extent of striatal ERK1/2 phosphorylation produced by the last drug dose was positively and strongly correlated with the AIMs scores recorded during the treatment period [33]. Levels of ERK1/2 activation in chronically l-DOPA treated nondyskinetic animals did not differ significantly from baseline values. Thus, the pronounced activation of ERK1/2 in DA-denervated striatal neurons provided a molecular counterpart to the induction of AIMs by l-DOPA [34]. Because an increased phosphorylation of ERK1/2 was also produced by acute l-DOPA treatment (which does not elicit significant AIMs), previous study suggested that the core signaling alteration associated with dyskinesia consists in an inability to desensitize the phospho-ERK1/2 response with repeated exposure to l-DOPA [35]. In present study, the phospho-ERK1/2 expressing of levodopa group and levodopa liposomes group were significantly higher than the saline control group and the sham-operated group. However, the phospho-ERK1/2 expressing of levodopa liposomes group was significantly lower than the levodopa group, which suggested that application of chitosan-coated levodopa nanoliposomes may reduces the different movement disorder in PD treatment, compared to ordinary levodopa tablets.

Many evidence highlights the importance of DARPP-32 as a critical component of signal integration in striatal projection neurons. Once phosphorylated, the DARPP-32 at Thr-34 inhibits protein phosphatase-1 (PP-1) activity [36], acting as a cAMP-dependent kinase inhibitor [37]. In fact, in the dorsolateral striatum, phosphorylation mechanisms play a physiological role in motor control, and the induction of both long-term depression (LTD) and long-term potentiation (LTP), two opposing forms of synaptic plasticity. Both forms of plasticity (LTP/LTD) are also critically linked to the activation of DA receptors, supporting the idea that in the striatum, a close interplay among DA receptors, DARPP-32 state phosphorylated/de-phosphorylated, and glutamatergic transmission might underlie the functional role of this structure in motor control and cognitive activities [38]. In present study, the phospho-Thr34 DARPP-32 expressing of levodopa group and levodopa liposomes group were significantly higher than the saline control group and the sham-operated group. However, the phospho-Thr34 DARPP-32 expressing of levodopa liposomes group was significantly lower than the levodopa group, which suggested that application of chitosan-coated levodopa nanoliposomes may reduces the different movement disorder in PD treatment, compared to ordinary levodopa tablets.

## Conclusion

The molecular adaptations produced by chronic l-DOPA treatment in indirect pathway neurons are poorly understood. Some recent studies performed on rats with 6-OHDA lesions have specifically addressed the relative localization of l-DOPA-induced changes in striatal gene expression to dynorphinergic or enkephalinergic neurons and have consistently found a selective upregulation of transcription factors and plasticity genes in the former population [39]. In present study, it is tempting to propose involvement of FosB/ΔFosB, phospho-ERK1/2, and phospho-Thr34 DARPP-32 in these effects [40]. And compared with ordinary levodopa tablets, chitosan-coated levodopa liposomes may be a more useful agent in reducing dyskinesias inducing for Parkinson disease.

## Methods

### Animals

Experimental procedures were carried out on 60 adult male Sprague–Dawley rats (Tongji Medical College Laboratory Animal Center of Huazhong University of Science and Technology, Wuhan, China) weighting 180–250 g corresponding to ~6 weeks of age. All procedures were performed minimizing animal discomfort and strain. Rats were maintained on a regular light–dark cycle (lights on at 10:00 a.m., lights off at 10:00 p.m.; room temperature 20–22 °C; and maximum three animals per cage) and were given food and water ad libitum before habituation to the behavioral paradigm. We employed a 6-OHDA-based parkinsonian rat model inducing abnormal involuntary movements (AIMs) comparable with LID observed in PD patients by chronic treatment with levodopa. The Institute Research Medical Ethics Committee of Huazhong University granted approval for this study.

### Unilateral 6-OHDA-lesion model of PD

Unilateral (left hemisphere) DA denervation was performed according to a standard protocol [41]. Briefly, rats were anaesthetized with 1.5–2.5 % isoflurane in oxygen and mounted on a stereotaxic instrument (Stoelting Co, Wood Dale, IL, USA). Body temperature was maintained at 37–38 °C with a heating pad (Stoelting Co) placed beneath the animal. After a subcutaneous injection of the local anesthetic bupivacaine, a midline scalp incision was made, and a hole (diameter of ~1.0 mm) was drilled in the skull on the left side. The neurotoxin (30-mM solution of 6-OHDA containing 0.03 % of ascorbic acid) was injected into the medial forebrain bundle (coordinates: 4.0 mm posterior of the bregma, 1.3 mm laterally of the midline, and 7.0 mm beneath the cortical surface). Injections of 3 μL of 6-OHDA were administrated through a 30-gauge cannula connected to a 10 μL Hamilton syringe over a period of 3 min. The injection of neurotoxin was preceded by a bolus of desipramine (25 mg/kg, intraperitoneally) to minimize the uptake of 6-OHDA by noradrenergic neurons. Two weeks later, an apomorphine-induced rotation test (0.05 mg/kg, subcutaneously) was performed to assess the severity of nigral lesions [41]. Animals performing at least 100 rotations opposite to the lesion site within 20 min from the apomorphine treatment were considered successfully lesioned and included in the study [42].

### Chitosan-coated levodopa nanoliposomes

We used the chitosan-coated levodopa nanoliposomes as previously described [40].

### Drug treatment and behavioral tests

6-OHDA-lesioned rats randomly divided into three groups (saline control group, n = 10; levodopa group, 20 mg/kg levodopa and 5 mg/kg benserazide, n = 20; levodopa liposomes group, 4.7 mL/kg 34.34 % chitosan-coated levodopa nanoliposomes PBS solution and 5 mg/kg benserazide, n = 20) and sham-operated rats (n = 10) intragastric administrated twice a day up to 21 days. Animals were placed individually in a Plexiglas box and their axial, limb, and orofacial AIMs were monitored daily between 10:00 a.m. and 04:00 p.m. for 5 min per animal at 1, 3, 6, 9, 12, 15, 18, 21 days after levodopa administration by two trained researchers blind to the treatment. AIMs were scored on a severity scale ranging from 0 to 4 (1 = occasional; 2 = frequent; 3 = continuous but interrupted by sensory distraction; and 4 = continuous, severe, not interrupted by sensory distraction) as previously described [43].

### Western blotting

An independent group of rats treated with either rosiglitazone (RGZ) (10 mg/kg) or vehicle (n = 8/group) were anesthetized with isoflurane on day 21 of levodopa treatment, 120 min after the last administration of levodopa, and sacrificed by decapitation. Brains were rapidly removed and frozen in cold 2-methylbutane (−50 °C). Brain coronal sections (1 mm) were cut in an ice-cold stainless steel mold using razor blades and the striatum dissected out and homogenized in a glass Potter–Elvehjem, homogenized in ice-cold lysis buffer (50 mM Tris–HCl, 100 mM NaCl, 0.1 % Triton X-100, 0.1 % SDS, 1 mM Na_3_VO_4_, 10 mM NaF, and 1 mM EDTA) and 1 % protease inhibitor cocktail (Sigma Chemical, St. Louis, USA), and centrifuged at 16,000*g* for 30 min at 4 °C. Equal amounts of protein (20 μg) were resolved by SDS-PAGE (10 %), transferred onto PVDF membranes (0.2 μm), and incubated for 1 h in 5 % fat-free milk in Tris buffer saline + 0.05 % Tween-20 (TBS-T buffer) at room temperature. Membranes were then incubated overnight at 4 °C using the following primary antibodies: FosB/ΔFosB, ERK1/2, p-ERK1/2, p-Thr43 DARPP-32, DARPP-32 (Santa Cruz, 1:1000), and β-actin (Sigma, 1:10, 000). After three 5-min washes in TBS-T, membranes were incubated with the appropriate secondary horseradish peroxidase-linked antibodies (Santa Cruz, 1:2000) for 60 min at room temperature. Protein bands were visualized using the ECL kit (Amersham, CE Healthcare, Buckinghamshire, England) followed by exposure to X-ray film. Band immunoreactivity was quantified by densitometry using NIH image software.

### Immunohistochemistry

The FosB/ΔFosB, ERK1/2 and p-ERK1/2 immunohistochemical analyzed by the ABC-peroxidase method on day 21 of levodopa treatment, 120 min after the last administration. The tissue and cells samples were prepared as frozen sections. After fixation with acetone and washing with PBS, the sections were incubated with a primary FosB/ΔFosB, ERK1/2 and p-ERK1/2 antibody (1:1000 dilution, Santa Cruz, USA) overnight at 4 °C, then incubated with a biotinylated secondary antibody (1:200 dilution) at room temperature for 1 h. After that, the ABC-peroxidase reagent (Vector, USA) was added for an additional 1 h. With the PBS washing and 3, 3-diaminobenzidine (30 mg dissolved in 100 mL Tris buffer containing 0.03 % H_2_O_2_) staining for 5 min, the samples rinsed in water and counterstained with hematoxylin. Total ten visual fields were examined randomly in each section under 200× magnification under the light microscope and the positive stained cells were counted in a total number of 500–1000 cells.

### Statistical analysis

Behavioral data and group comparison of dyskinesia intensity scores were analyzed by repeated measures (ANOVA) and relevant differences between groups were analyzed pairwise by Bonferroni post hoc test. Global AIMs comparison between two groups was performed by using Student’s t test. The null hypothesis was rejected when *P* < 0.05.

For analysis of WB and IHC quantification data, Student’s t test was used (minimum level of significance *P* < 0.05) provided by GraphPad PRISM 5 software.
